# Effect of Trace Amounts of Chloride on Roughening of Au(111) Single-Crystal Electrode Surface in Sulfuric Acid Solution during Oxidation–Reduction Cycles

**DOI:** 10.1021/acselectrochem.4c00226

**Published:** 2025-03-03

**Authors:** Saeid Behjati, Marc T. M. Koper

**Affiliations:** Leiden Institute of Chemistry, 4496 Leiden University, PO Box 9502, 2300 RA Leiden, The Netherlands

**Keywords:** Au(111), chloride, hydrochloric acid, sulfuric acid, roughening, oxidation−reduction cycles, EC-STM, in situ scanning probe microscopy

## Abstract

This study investigates the impact of varying trace-level chloride ion concentrations on the roughening of a Au(111) electrode during oxidation–reduction cycles (ORCs) in 0.1 M sulfuric acid by in situ scanning tunneling microscopy (STM). At the higher chloride concentration (50 μM), rapid dissolution of Au atoms and step line recession are observed in the recorded in situ STM images. The high surface mobility of Au atoms resulted in a lack of detectable vacancy islands in the images with minimal changes in cyclic voltammograms (CVs) and the complete absence of nano-island formation, which is observed in pure sulfuric acid. At moderate concentration (10 μM), the dissolution rate decreased substantially, so the initial step lines are still distinguishable after the 200 ORCs. The lower surface mobility leads to the formation of vacancy islands in the terraces, and these newly formed step sites give rise to additional peaks in the CVs. At the lowest concentration (1 μM), nano-island formation is observed. However, inhomogeneous chloride adsorption (showing as darker areas in the EC-STM images) on the sample at high enough anodic potential (0.9 V) led to previously unreported behavior, showing very inhomogeneous roughening, with parts on the surface showing reduced Au atom mobility and minimal changes even after 200 ORCs.

## Graphical abstract

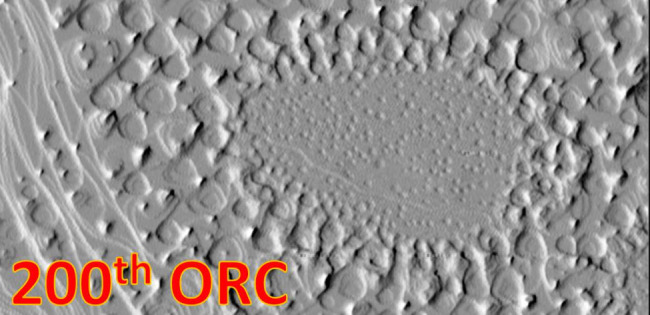

## Introduction

The excellent chemical stability of Au leads to its extensive use in various electrochemical conditions. To understand the detailed surface chemistry of Au, Au single crystals have been studied both in ultra-high vacuum (UHV)^1,2^ and aqueous electrochemical environments.^3–5^ Au oxidation has been studied in sulfate- and perchlorate-containing electrolytes.^4,6–8^ In sulfate-containing electrolytes, at sufficiently positive potential, sulfate anions will form an ordered adlayer on Au(111),^9^ which influences the onset potential for surface oxidation by the blocking effect of adsorbed anions. The oxidation starts with electroadsorption of OH^–^:

$$\mathrm{Au}+H_{2}O\to \mathrm{Au-}\mathrm{OH}+H^{+}+e^{-}$$ 1

At higher potentials, the formation of Au­(OH)_3_, AuOOH, and Au_2_O_3_ has been suggested.^10^ Successive oxidation–reduction cycles (ORCs) applied to the Au(111) single-crystal electrode in sulfuric acid creates a highly roughened surface with long-range nanopatterns.^3,11^ The surface roughness is caused by oxide formation pushing out Au surface atoms, which form adatom and vacancy islands after subsequent reduction.^12^ If the electrode is paused within the double layer potential window, the surface mobility of the Au surface atoms will smoothen the surface, making the final roughness very sensitive to the time spent at different potentials.^11^ It is well known that the electrochemical behavior of Au surfaces is very sensitive to trace amounts of chloride ions present in the electrolyte.^13,14^ Both one and three electron oxidation processes have been proposed for Au dissolution at positive potentials:^14,15^

$$\mathrm{Au}+4{\mathrm{Cl}}^{-}\to {{\mathrm{AuCl}}_{4}}^{-}+3e^{-}$$ 2

$$\mathrm{Au}+2{\mathrm{Cl}}^{-}\to {{\mathrm{AuCl}}_{2}}^{-}+e^{-}$$ 3

Thus, in the presence of chloride, Au surface oxidation and Au dissolution occur simultaneously at anodic potentials. At a high concentration of chloride (1 mM) in perchloric acid, anisotropic dissolution of Au was reported.^16^ Moreover, apart from causing dissolution during the ORCs, trace amounts of chloride can enhance the step motion and prevent roughening.^17^ Investigation of step dynamics on Au(111) in chloride-containing electrolytes showed that the specifically absorbed chloride can change the dominant mass transport mode from terrace diffusion to edge diffusion.^18^

In this study, we conduct an in-depth in situ electrochemical scanning tunneling microscopy (EC-STM) study of the oxidation–reduction cycling of a Au(111) electrode with varying chloride ion concentrations in 0.1 M sulfuric acid. We show how these changes in electrolyte composition influence surface evolution during the ORCs, particularly the role of chloride in the dissolution rate, roughening process, and Au surface atom mobility. Notably, at the lowest chloride concentration, we show how chloride appears to amplify the inhomogeneity of the surface by roughening certain parts of the surface, while other parts remain unaltered.

## Experimental Section

### EC-STM Measurements

The electrochemical scanning tunneling microscopy (EC-STM) images were captured using a custom-built instrument developed at the Leiden Institute of Chemistry (LIC) at Leiden University. More information about the instrument can be found in our previous paper.^11^ The tips were fabricated from a platinum/iridium wire (90/10) using the pulling-cutting method. To minimize additional faradaic currents at the tip, a layer of hot melt adhesive (EVA-copolymer, synthetic resin, wax, and stabilizer, Brand: C.K.) was applied, leaving only the apex of the tip exposed. A disk-shaped single-crystal electrode Au(111) (10 mm in diameter) with a Au wire welded to the back was used as the working electrode (WE). The crystal was cut with a precision of 0.1° and polished to a roughness of 30 nm by the Surface Preparation Laboratory (SPL) in the Netherlands. Before each measurement, the Au(111) sample was annealed using a butane flame torch until it turned orange, maintained for 5 min, and then cooled in air above ultrapure water to prevent contamination of the sample surface. A high-purity Au wire was used as the counter electrode (CE), and a reversible hydrogen electrode (RHE, Hydroflex, Gaskatel) was used as the reference electrode (RE). Images were recorded in constant current mode with a current set point ranging from 50 to 150 pA and a tunneling bias of 10 to 20 mV. The tip was retracted hundreds of nanometers during the CV recording. Throughout the experiment, the EC-STM chamber was purged with ultra-high-purity argon gas to minimize the dissolution of oxygen or other gases into the EC-STM cell. Despite these efforts, there is still the possibility that a trace amount of oxygen is present. We expect trace oxygen or oxygen reduction on the Au surface to be only a very minor, if at all, disturbance to the surface structure.

### Electrochemical Cell and Electrolyte

A custom-made Pyrex glass cell was utilized for standard electrochemical experiments. All glassware and plastic components were thoroughly cleaned by soaking in a permanganate solution (0.5 M sulfuric acid and 1 g/L potassium permanganate) for a minimum of 12 h before each experiment. After rinsing with Milli-Q water, the components were treated with a diluted piranha solution (3:1 mixture of sulfuric acid (H_2_SO_4_) and hydrogen peroxide (H_2_O_2_), diluted with water) to eliminate manganese oxide and permanganate residues. To remove any remaining diluted piranha, all parts were boiled at least five times. The electrolyte, composed of H_2_SO_4_ (96% Suprapur Sigma-Aldrich) and HCl (30% Suprapur Sigma-Aldrich), was prepared using ultra-high-purity (UHP) Milli-Q water (resistivity > 18.2 MΩ·cm) and degassed with ultra-high-purity argon gas for at least 30 min. All measurements were conducted at room temperature (*T* = 293 K).

## Results and Discussion

The roughening process of Au(111) in 0.1 M sulfuric acid has been studied well in previous works.^3,11^ In order to be able to compare and clearly distinguish the role of chloride during the ORCs, the sulfuric acid concentration was kept constant in all experiments, and the chloride concentration was varied between 1, 10, and 50 μM.

### Oxidation–Reduction Cycles of Au(111) in 0.1 M H_2_SO_4_ and 50 μM HCl

The first experiment was devoted to the highest chloride concentration, i.e., 50 μM. Figure 1a shows the sample surface in 0.1 M H_2_SO_4_ and 50 μM HCl at 0 V vs RHE just after the Au(111) electrode was annealed. The differential image shown on the bottom-right of the image gives a better contrast to see the Au(111) reconstruction pattern. Moreover, some step lines can be seen, which will be useful since we expect some dissolution from step lines after applying CVs. Thus, having these step lines in the scan area can help to evaluate step line activity. The top half of Figure 1b is recorded at 0.6 V, and the bottom half (below the dashed line) is recorded at 0.7 V vs RHE. The formation of small adatom islands is clear at 0.7 V. After applying 0.8 V vs RHE, Figure 1c was recorded, which shows some large monoatomic islands and one vacancy island. The most likely reason for these large islands is the increased mobility of the Au surface atoms in the presence of (adsorbed) chloride,^18,19^ and the Au adatom islands have grown (Ostwald ripening) before image recording. The shape and size of the islands are smaller and more triangular in pure sulfuric acid, but with chloride, their size increases, while their shape is more circular. After five consecutive ORCs with 0.9 and 1.7 V as the lower and upper potential limits (scan rate of 50 mV s^–1^), Figure 1d was recorded. Comparing this frame with the previous one, two main differences can be noted: all of the adatom islands have dissolved, and step line recession has taken place, as most clearly evidenced by the disappearance of the narrow terrace. The red dashed line represents the step lines before applying 5 ORCs (Figure 1c). After 10 ORCs, the image in Figure 1e shows more step line recession but no vacancy islands. Two possible mechanisms can be considered for this behavior. Either the dissolution of Au atoms only takes place at the step edges and the terraces stay unchanged, or there is also some dissolution taking place on the terraces, but the mobility of the atoms is so high that the vacancy islands effectively move quickly^20^ until they are captured by the step lines and vanish.^21^ The latter case is more probable since some large vacancy islands appear after 50 ORCs, as illustrated in Figure 1f. This indicates that Au dissolution is also taking place from the terraces. The step line recession is now so severe that the initial step lines cannot be observed anymore. Figure 1g–i shows the surface development after 100, 150, and 200 ORCs, respectively, confirming that a higher cycle number leads to more dissolution and continuing step line recession.

**Figure 1:**
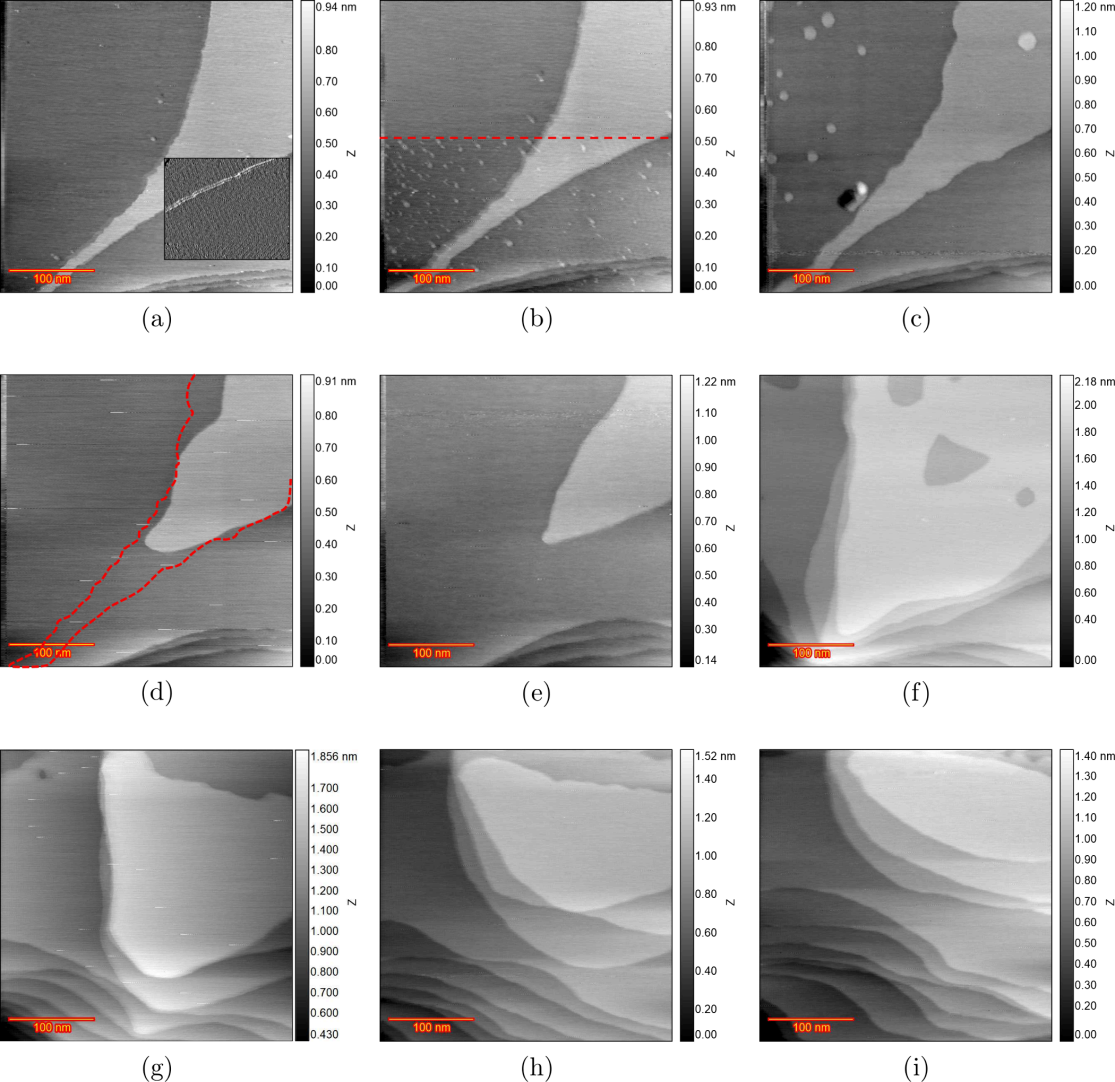
EC-STM images (350 × 350 nm) of Au(111) in 0.1 M H_2_SO_4_ and 50 μM HCl. (a) Sample surface at 0 V vs RHE just after annealing. (b) Top half is recorded at 0.6 V, and from the red arrow downward, the EC voltage changed to 0.7 V. (c) Fully lifted reconstruction at 0.8 V. (d) After *n* = 5, (e) *n* = 10, (f) *n* = 50, (g) *n* = 100, (h) *n* = 150, and (i) *n* = 200 ORCs.

From the above experimental results, we conclude that with a (relatively) high concentration of chloride in solution, surface oxidation and subsequent reduction do not lead to roughening (formation of adatom and vacancy islands). The dominant process on the surface is Au dissolution by reactions 2 and 3. Moreover, the mobility of the chloride-covered Au atoms is so high that vacancy islands are also highly mobile, and the surface development at higher ORC numbers is mostly taking place by step line recession.

Figure 2a presents the recorded CVs on Au(111) in 0.1 M H_2_SO_4_ and 50 μM HCl with a scan rate of 50 mV s^–1^ in the potential window of 0.9 V to 1.7 V vs RHE. The first cycle (in blue) does not differ substantially from the last cycle (in red). As is evident from the EC-STM images, at high chloride concentrations, the surface roughness does not change very much (mainly dissolution and step line recession), as confirmed by the essentially identical CVs over 200 cycles. The main anodic and cathodic peaks are at 1.55 and 1.19 V vs RHE, respectively. The small anodic current peak at 1.13 V seems to be correlated to chloride adsorption since this peak disappears for lower concentrations of chloride. The small cathodic peaks at 1.09 V can be correlated to the desorption of chloride and minor redeposition of the dissolved Au atoms. Figure 2c shows the calculated charge density for both oxidation and reduction peaks as a function of the cycle number. Except for the few first cycles, the charge density shows a plateau, in line with the absence of roughening. The large difference between the oxidation and reduction charge density is due to the Au dissolution process.

**Figure 2:**
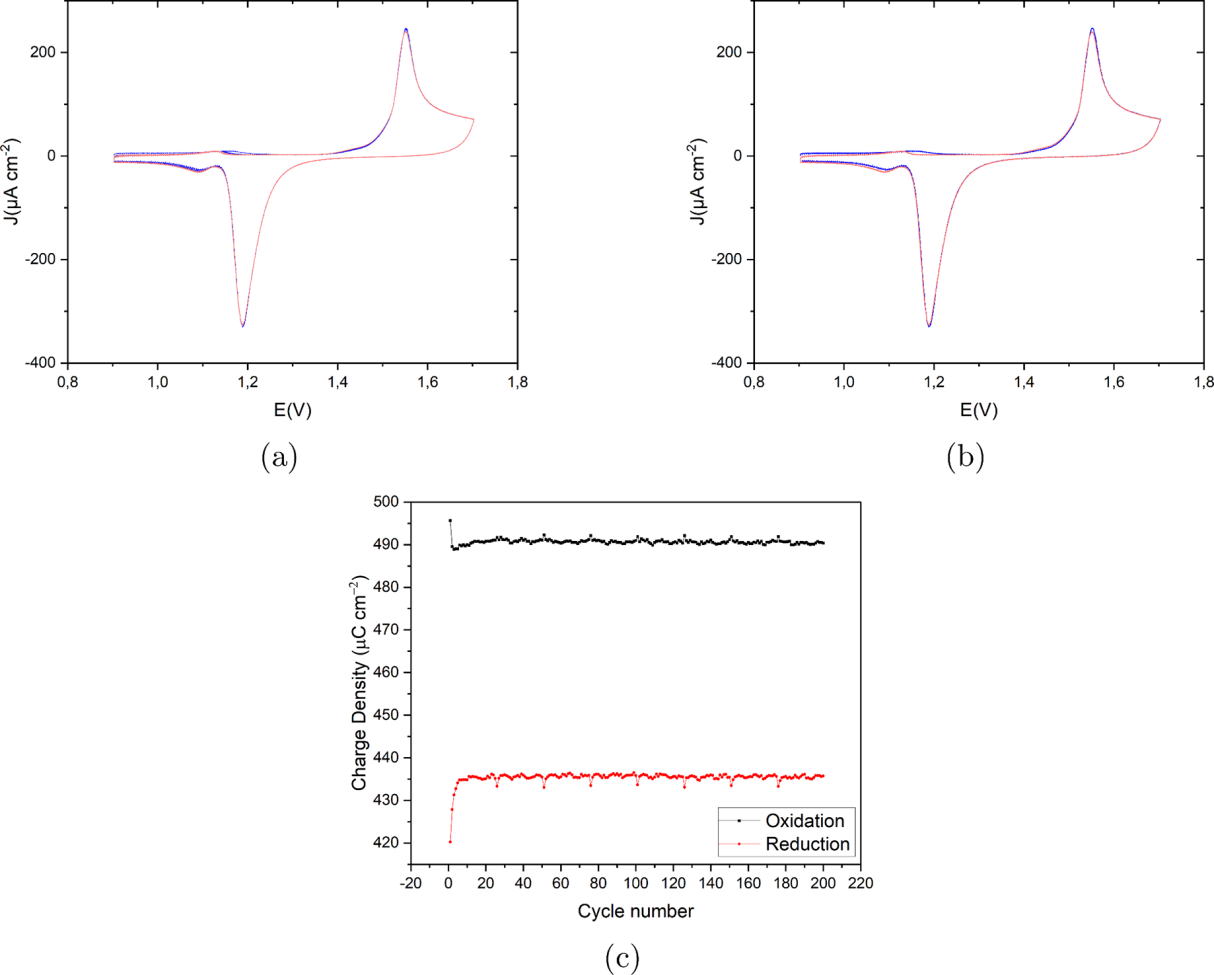
(a) Cyclic voltammograms of the consecutively applied 200 ORCs on Au(111) in 0.1 M H_2_SO_4_ and 50 μM HCl with a scan rate of 50 mV s^–1^ vs RHE. The color spectrum ranges from blue for the first cycle to red for the last cycle. (b) First and last cycle in blue and red, respectively, for a better representation. (c) Calculated oxidation (black) and reduction (red) charge density (μC cm^–2^) vs cycle number for the CVs shown in (a).

### Oxidation–Reduction Cycles of Au(111) in 0.1 M H_2_SO_4_ and 10 μM HCl

For the next experiment, the concentration of hydrochloric acid was reduced to 10 μM. This change can help to pinpoint the role of trace levels of chloride in the surface evolution over many ORCs. Figure 3a shows the differential image of the pristine surface at 0 V vs RHE after annealing, which shows herringbone reconstruction on the large terraces. There are some adatom and vacancy islands in the bottom-left part, and these defects have been observed in many previous experiments showing that the annealed sample (with the corresponding method) is not flawless. The potential is then increased to the point that the reconstruction lifting process initiates. It is known that the lifting of reconstruction happens in several stages.^22^ Figure 3b shows the differential image recorded at 0.7 V vs RHE. Some small monoatomic islands show up at the very top of the image, which indicates the lifting of the reconstruction at that spot. However, the compact herringbone can still be seen in the middle and bottom parts, which indicates the local charge density is not high enough to initiate the lifting. Also, there is a spot between the aforementioned areas indicated with red spheroids which shows some distorted reconstruction, which can be considered as an intermediate stage. This suggests that even at the same potential, different areas can exhibit slightly different behaviors concerning surface reconstruction. At 0.8 V vs RHE, the reconstruction is fully lifted, as is shown in Figure 3c, since there is no sign of herringbone and the entire surface is covered with monoatomic islands. The island density is lower on the terraces near the upward step lines, and this can be due to the capturing of the atoms/islands by the step line. Regarding the island shape, no preferred step type/direction is observed, and islands appear circular. The island size is less than that in the captured frame in Figure 1c for higher chloride concentration. Figure 3d was recorded after 5 ORCs within the same potential window. The number of islands reduced substantially, while their size increased, which can be caused by Ostwald ripening. Moreover, some slight changes in the step lines can be observed (i.e., smoothening of the rough step line in the bottom-left corner). The red dashed line shows the same step in the previous frame. In 50 μM chloride, no adatom islands were left at this cycle number (Figure 1d), and the step line receded much more (by comparison of the dashed lines and the new step lines after 5 ORCs), which underscores the role of chloride in the surface evolution. After 10 ORCs, Figure 3e shows only five adatom islands and some more recession at the step line (e.g., see the step in the bottom-left). After 20 ORCs, the dissolution of all of the adatom islands took place, simultaneously with the formation of vacancy islands, as shown in Figure 3f. Figure 3g was taken after 50 ORCs and shows larger vacancy islands with a few adatom islands in the bottom part. Those islands can be related to the redeposition of dissolved Au atoms during the negative-going voltage sweep. Panels h and i of Figure 3 show the result after 100 and 200 ORCs, respectively. It is clear that the higher number of ORCs causes more Au dissolution, causing the formation of new vacancy islands and recession of the step lines.

**Figure 3:**
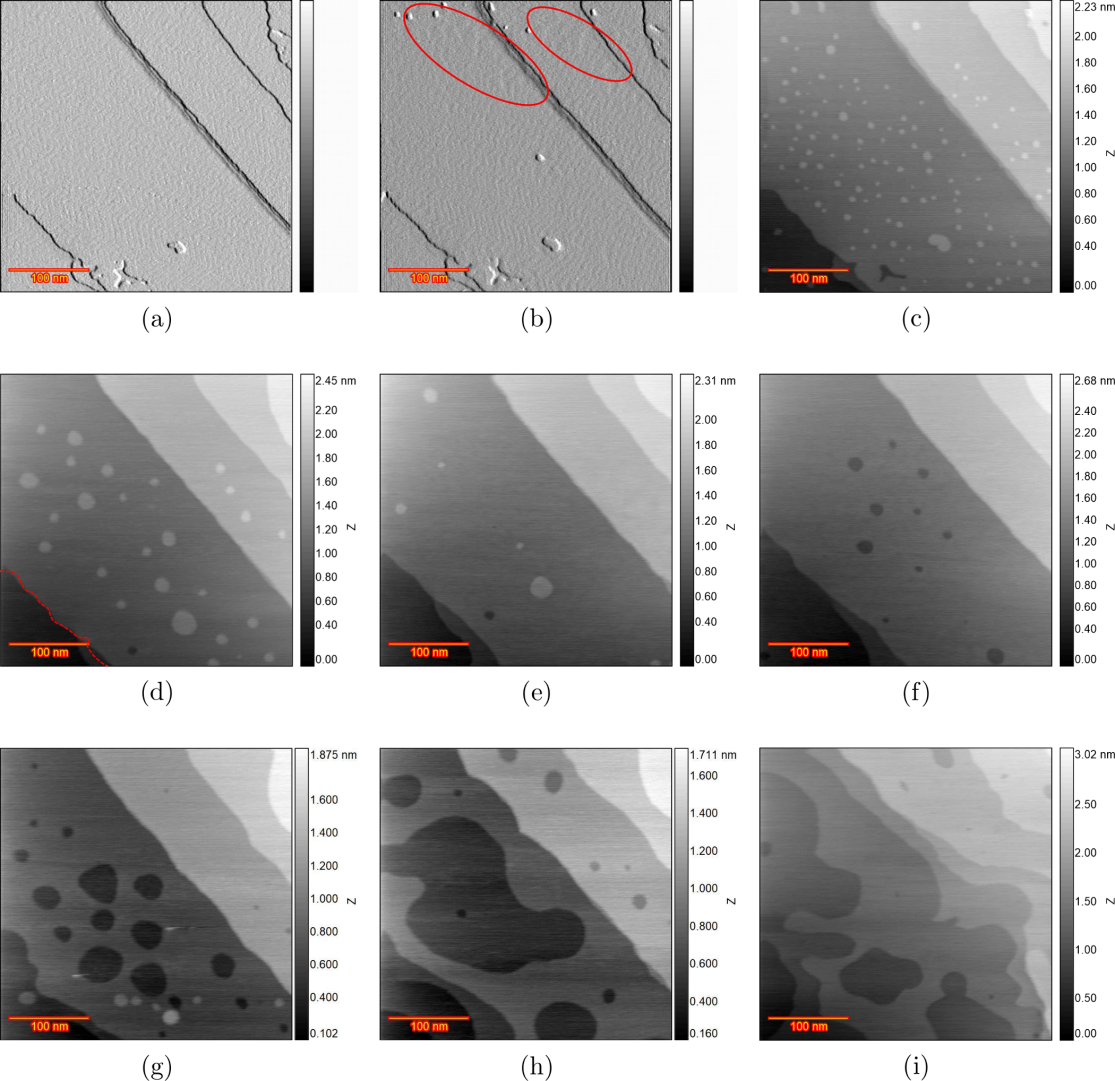
EC-STM images (350 × 350 nm) of Au(111) in 0.1 M H_2_SO_4_ and 10 μM HCl. (a) Differential image of the pristine surface at 0.6 V vs RHE after thermal annealing. (b) Differential image of partially lifted reconstruction at 0.7 V vs RHE. (c) Fully lifted reconstruction at 0.8 V. (d) After *n* = 5, (e) *n* = 10, (f) *n* = 20, (g) *n* = 50, (h) *n* = 100, and (i) *n* = 200 ORCs.

Figure 4a presents the recorded CVs of Au(111) in 0.1 M H_2_SO_4_ and 50 μM HCl with a scan rate of 50 mV s^–1^ in the potential window of 0.9 V to 1.7 V vs RHE. The first cycle (in blue) to the last cycle (in red) shows only slight changes. Specifically, the amplitude of the main anodic peak at 1.63 V reduces and shifts slightly to a higher potential at higher ORC numbers, and a small anodic peak emerges at 1.54 V with its amplitude increasing with the ORC number. We expect this peak to be related to oxidation at the step sites. The main cathodic peak is located at 1.15 V with no shoulder peaks for the first cycle. With more cycles, another peak appeared at 1.18 V, very close to the main cathodic peak, as well as a small shoulder at 1.11 V (indicated with an arrow in Figure 4b). The small cathodic shoulder peak at 1.11 V is likely correlated to the redeposition of dissolved Au atoms since the concentration of dissolved Au atoms in the diffusion layer is initially very low, but after more cycles, it can show up. Figure 4c shows the calculated charge density for both oxidation and reduction peaks as a function of ORC number. After 20 ORCs, there is a linear decay for both charge densities, and as expected, the reduction charge is lower than the oxidation charge. The difference between oxidation and reduction charge is less than in the experiment with 50 μM, showing that the extent of Au dissolution is related to the chloride concentration.

**Figure 4:**
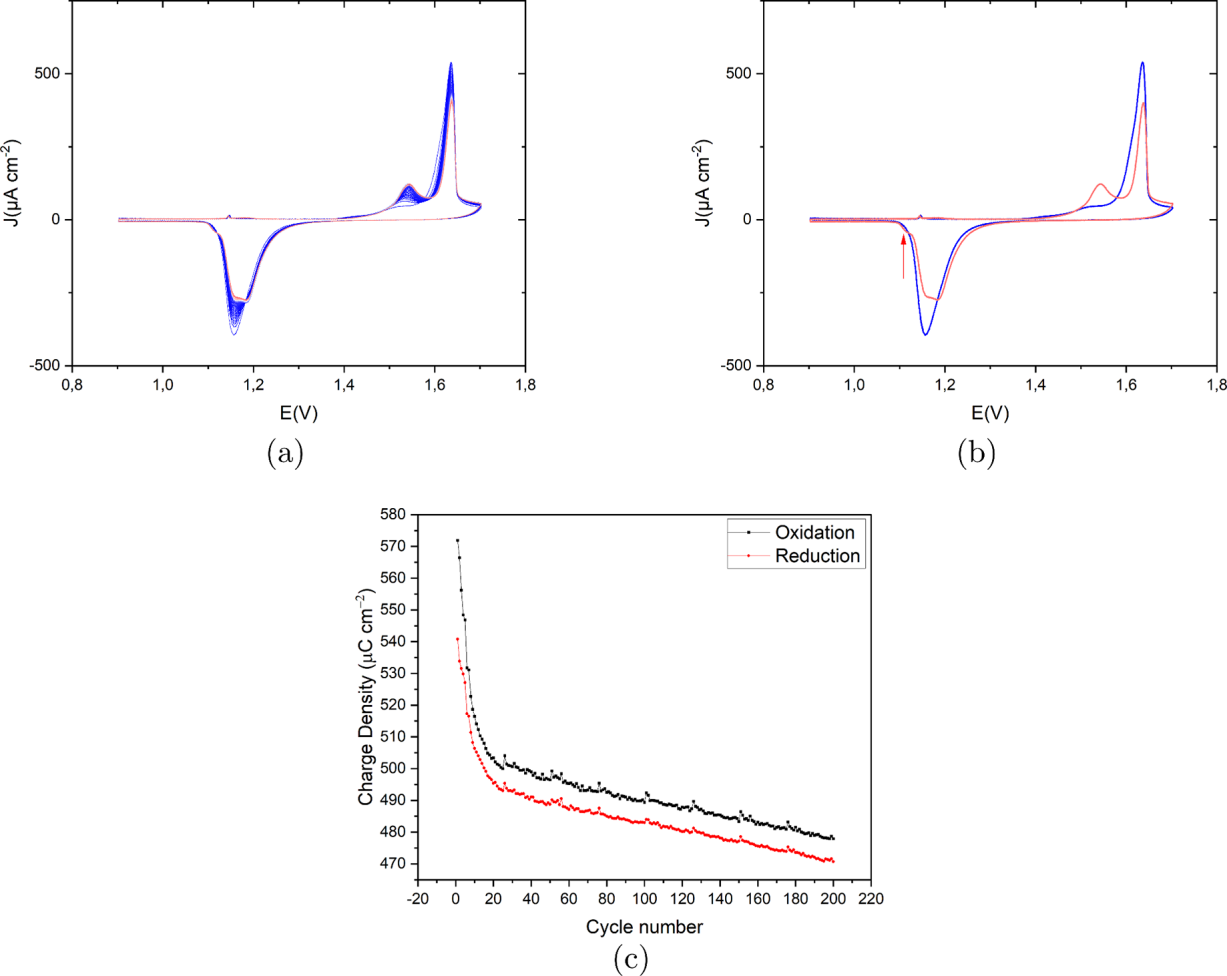
(a) Cyclic voltammograms of the consecutively applied 200 ORCs on Au(111) in 0.1 M H_2_SO_4_ and 10 μM HCl with a scan rate of 50 mV s^–1^ vs RHE. The color spectrum ranges from blue for the first cycle to red for the last cycle. (b) First and last cycle in blue and red, respectively, for a better representation. (c) Calculated oxidation (black) and reduction (red) charge density (μC cm^–2^) vs cycle number for the CVs shown in (a).

### Oxidation–reduction Cycles of Au(111) in 0.1 M H_2_SO_4_ and 1 μM HCl

For the final experiment, the chloride concentration was reduced to 1 μM. Figure 5a shows the pristine Au(111) surface at 0 V vs RHE after annealing: there are only two terraces with a curved step line in the scanning area. Figure 5b contains two parts, which are separated by a red dashed line: the top half is recorded at 0.8 V vs RHE and the bottom half at 0.9 V. The top half shows a vacancy island surrounded by adatom islands formed on the top-right part of the image. This feature appeared at 0.6 V and can be related to having some contaminations/defects in the sample, which leads to this early island formation. Other than that, no substantial change is observed in the top half. On the other hand, in the bottom half, the reconstruction is lifted. It is important to notice the darker areas that appeared in the bottom part, along with the lifting of the reconstruction. The darker areas suggest a different local work function, which can alter the local tunneling current magnitude. Since all the images are recorded in constant current mode, the feedback system will compensate for that change by adjusting the tip height. Thus, the change in the work function can be seen as some depressions in the surface. These changes in height would then not correspond to a real topographic feature of the sample. The influence of the adlayer on the topographical image of Cu(111) has previously been observed as a lower height of the terrace (ca. 0.05 nm) and assigned to the lower conductivity of the Cu terrace covered by adsorbates.^23^ Although we do not expect surface oxidation at 0.9 V, similar patches have been observed on Au(111) when an oxide layer is forming.^24^ This implies that any factor influencing the work function can lead to comparable results. What causes the local work function changes, with presumably a correspondingly different anion adsorption, remains unfortunately unresolved. Interestingly, the island size in those darker areas in Figure 5b is smaller than at other locations. This suggests that the composition of the interface in these regions must be different from elsewhere. Since this behavior is not observed in a pure sulfuric acid solution, the emergence of these areas should be attributed to the presence of a trace level of chloride anions and their influence on the surface chemistry. The next frame in Figure 5c is recorded at 0.9 V vs RHE; the large dark area is now located in the center of the image. Moreover, there are some new small dark spots at the bottom half, which were not (yet) observed in the previous image. This can be caused by the tip effect or by some extra adsorption/desorption of anions at this potential. The former reason is less probable since the STM scan line is from left to right. In case of a tip effect on the double layer composition of the darker areas, enlargement of the dark areas in that direction would be expected; however, there are many new spots, spatially separated, without a clear direction. The second reason would imply a certain slowness in the surface chemistry, which may be related to the very low chloride concentration. Figure 5d was recorded after 5 ORCs. It is obvious that the surface response on the dark areas is different from the rest of the sample surface since it seems that neither roughening nor dissolution is taking place there. The result for other spots is very similar to the experiment in pure sulfuric acid,^11^ which shows normal roughening caused by the place exchange mechanism during ORCs, leading to rounded-edge triangular islands. This observation implies that there is a direct correlation between the dark regions and the unroughened regions after the ORCs. Figure 5e shows the result after 10 ORCs. Surprisingly, even up to this cycle number, the darker areas stayed pristine. However, at *n* = 50, shown in Figure 5f, those areas are slowly shrinking, which can be caused by the tip effect or by the ORCs. At higher cycle numbers of 100, 150, and 200 (Figure 5g–i), the surface becomes more homogeneously roughened, but some differences can still be observed.

**Figure 5:**
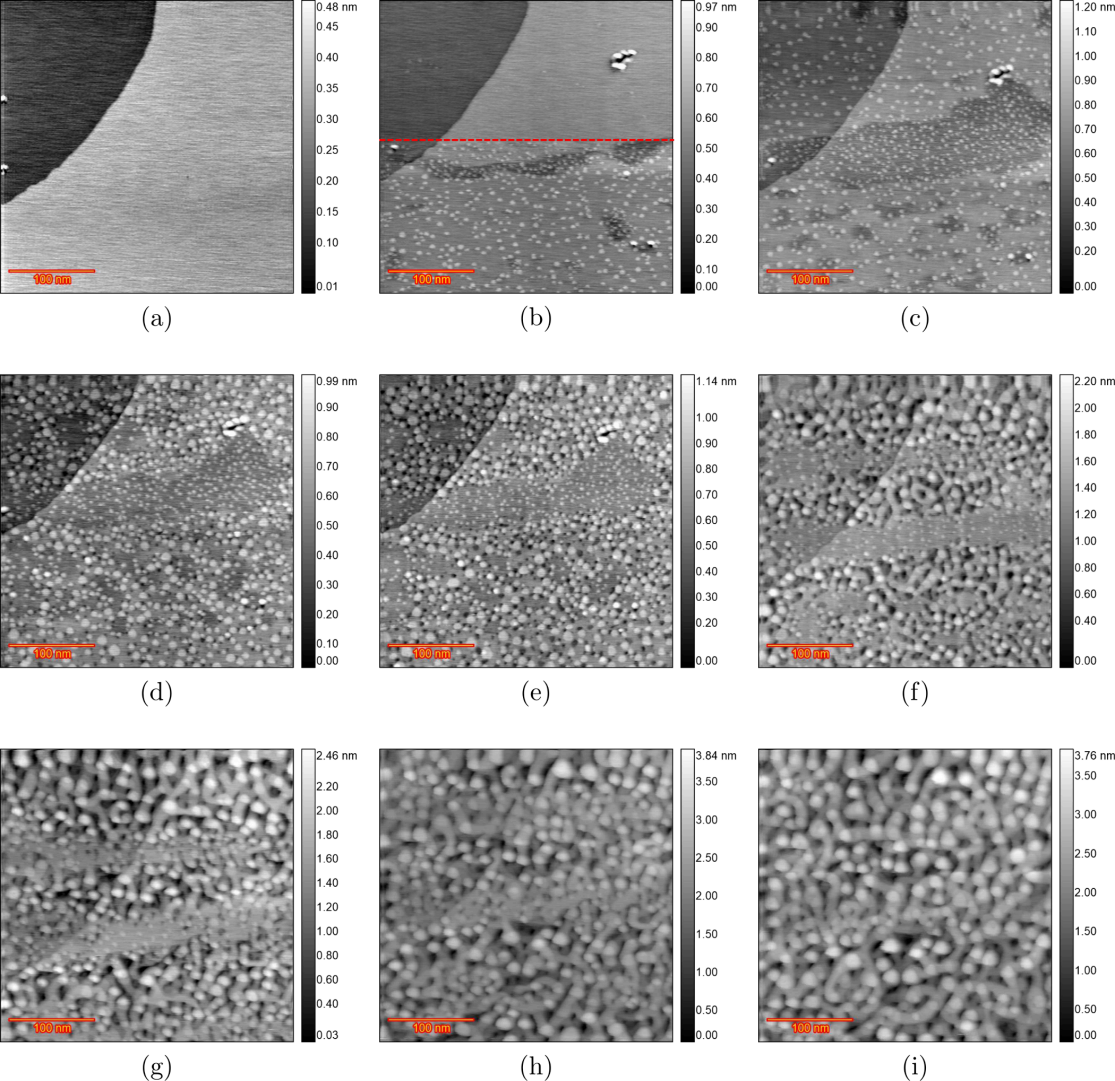
EC-STM images (350 × 350 nm) of Au(111) in 0.1 M H_2_SO_4_ and 1 μM HCl. (a) The pristine surface at 0 V vs RHE after annealing. (b) The top half is recorded at 0.8 V, and from the red dashed line downward, the EC voltage changed to 0.9 V. (c) Fully lifted reconstruction at 0.9 V with the emergence of some darker spots. (d) After *n* = 5, (e) *n* = 10, (f) *n* = 50, (g) *n* = 100, (h) *n* = 150, and (i) *n* = 200 ORCs.

The zoomed-out frame recorded after 200 cycles is shown in Figure 6a. The initial scan area is located almost at the center of the image, and the comparison with the rest of the image suggests that less roughening took place in the initial scan area. Thus, locations with a smaller/no contribution of chloride anions behave very similarly to pure sulfuric acid.^11^ A further zoomed-out image is shown in Figure 6b. The red square indicates the initial scan area of Figure 5. Some areas remain pristine, as highlighted by the yellow circles. Figure 6c shows the same image as Figure 6b in differential mode for better visualization. Figure 6d shows the zoomed-in image of the unroughened areas in differential mode. Small adatom islands, which are the result of the lifting of the reconstruction, are noticeable within these areas, and a step line is also visible. The transition area between roughened and unroughened areas contains smaller islands, which suggests that the border between the two areas is not abrupt or that islands grow by adatoms (generated by a place exchange mechanism in the roughened region) diffusing from all directions, which becomes discontinuous at the borders.

**Figure 6:**
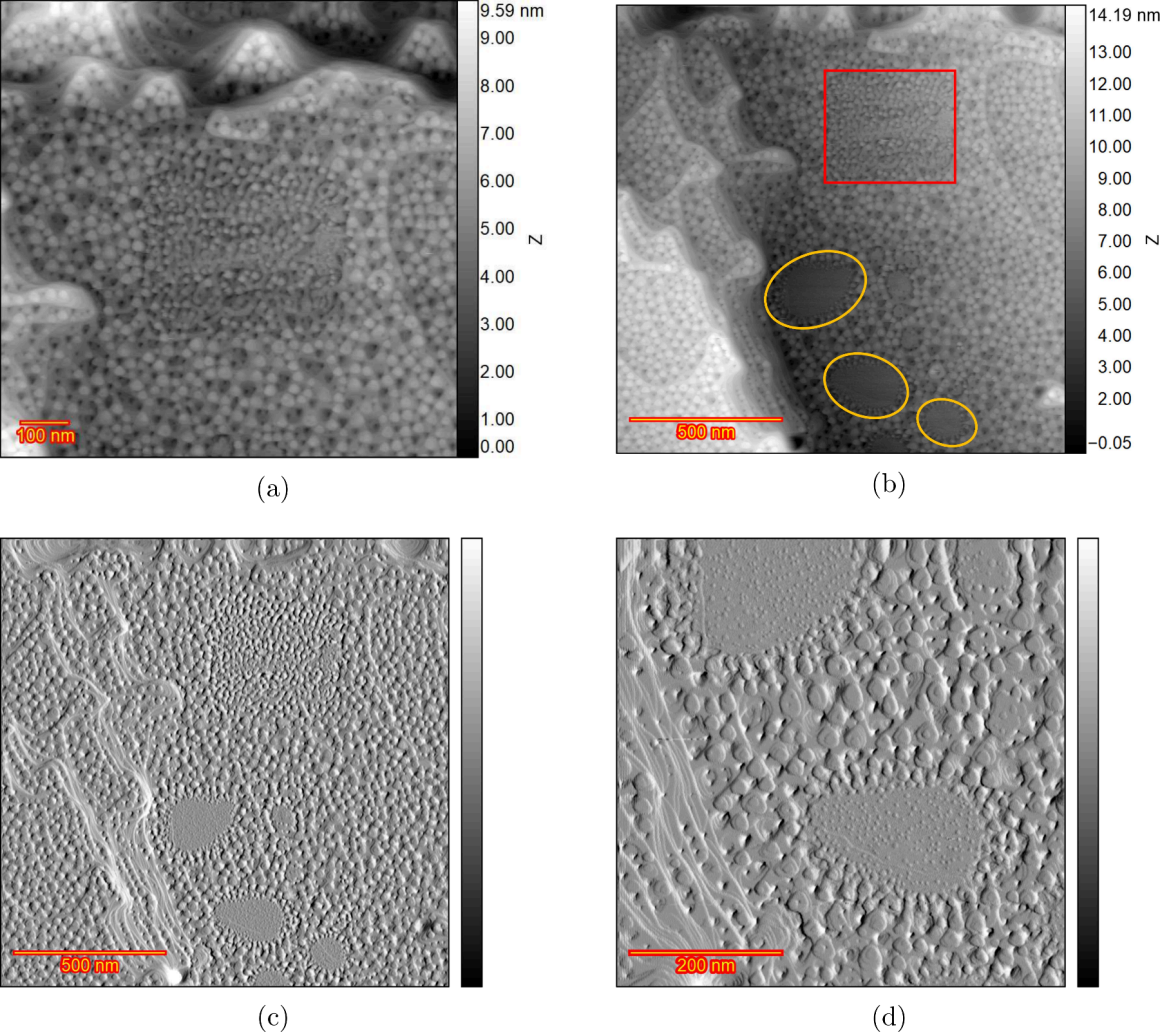
Au­(111) in H_2_SO_4_ and 1 μM HCl after 200 ORCs. (a) Large scan area which includes the initial scan area at the center. (b) Further zoomed-out frame which contains the initial scan area and some spots with no roughening. (c) The differential image of the frame shown in (b). (d) Zoomed-in image of the spots with no roughening.

Figure 7a presents the recorded CVs of Au(111) in 0.1 M H_2_SO_4_ and 1 μM HCl with a scan rate of 50 mV s^–1^ in the potential window of 0.9 V to 1.7 V vs RHE. The first cycle (in blue) to the last cycle (in red) shows slight changes only in the anodic peak shape, while the reduction peak shape is almost constant. The amplitude of the main anodic peak at 1.62 V reduces and shifts to a slightly lower potential for the first five cycles; for higher cycle numbers, the amplitude shifts to a higher current density and potential. Moreover, a small and broad anodic peak emerges at 1.42 V, its intensity increasing with cycle number. The main cathodic peak is located at 1.16 V, with a very slight reduction over cycles (Figure 7b shows the first and last cycle). Figure 7c shows the calculated charge density for both oxidation and reduction peaks with cycle number, showing a quick lowering and subsequent rising of the charge, followed by a slow decay.

**Figure 7:**
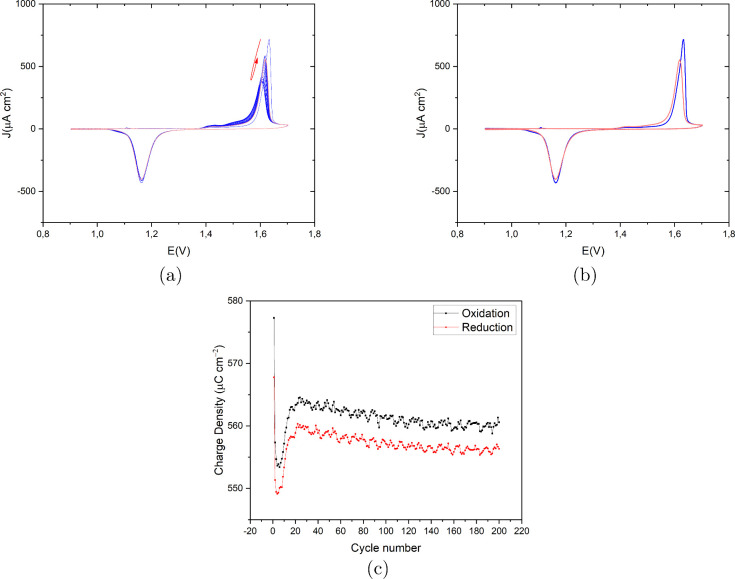
(a) Cyclic voltammograms of the consecutively applied 200 ORCs on Au(111) in 0.1 M H_2_SO_4_ and 1 μM HCl with a scan rate of 50 mV s^–1^ vs RHE. The color spectrum ranges from blue for the first cycle to red for the last cycle. The arrows show the trajectory of the oxidation peak. (b) First and last cycle in blue and red, respectively, for a better representation. (c) Calculated oxidation (black) and reduction (red) charge density (μC cm^–2^) vs cycle number for the CVs shown in (a).

From the above results, it is clear that chloride-induced Au dissolution plays an important role in the surface development of Au electrodes during oxidation–reduction cycles. Figure 8 illustrates the effect of chloride concentration by a histogram showing the difference in oxidation and reduction charge densities for all of the cycles for the three chloride concentrations. The value of the difference between the oxidation and reduction charge density at the maximum densities for 50, 10, and 1 μM chloride is 54.96, 7.21, and 3.94 μC cm^–2^, respectively. Increased chloride concentrations result in a greater charge difference, underscoring the critical role of chloride ions in facilitating Au dissolution. At a concentration of 50 μM, chloride not only promotes a higher dissolution rate but also enhances surface atom mobility. That explains why more step line recession and fewer vacancy islands were captured in STM images, as the chloride coverage on the surface is at the highest level at this concentration. As the chloride concentration decreases to 10 μM, the reduced mobility of Au atoms allows the capturing of vacancy islands. At the lowest HCl concentration (1 μM), some regions outside the initial scanned area in Figure 6b remained pristine even after 200 cycles, indicating that tip effects were primarily responsible for the observed roughening in darker regions by disturbing the interfacial layer in repeated surface scans after ORCs. The tip effect is only important in 1 μM HCl because, under these conditions, any inhomogeneities in the surface properties appear to become amplified and hence become susceptible to disturbances from the tip. In contrast, this disturbance was not important in other experiments due to the greater homogeneity of the surface. Formation of the areas with different levels of roughening is likely due to the inherent inhomogeneity in the surface, which would be amplified by the chloride-containing electrolyte, for instance, by having a different adsorbed adlayer. Perhaps there is a threshold (local) chloride concentration required to form an adlayer containing chloride, which could lead to this unexpected amplifying behavior. It seems that in those spots that are not roughened, the Au atom surface mobility is very low since the small adatom islands did not go through ripening steps. Reduction in surface mass transport rates is expected in areas with different absorbed layers. Previous STM studies of Au(111) in UHV studied the effect of sulfur and oxygen, the lifting of the reconstruction by sub-monolayer S coverage, and the adlayer structure of S adatoms.^25–28^ Adsorbed sulfur on Au(111) was shown to have an effect on the enhancement of the decay rate of monoatomic Au islands.^29^ It has been proposed that chemisorbed species can improve metal surface dynamics by forming metal-additive complexes, which can lead to easier mass transport across the surface on Cu and Ag samples.^30,31^ Accordingly, sulfate and other adsorbates can modify electrochemical reactions, Au atom mobility, the formation energy of step lines and kinks, and other related processes in various crystallographic orientations. In addition, adsorbed sulfate presumably inhibits the adsorption of impurities. These influences collectively determine the dynamics of roughening, the shape of islands, and the final surface roughness after the ORCs. Moreover, neither oxidation (place exchange mechanism) nor Au dissolution appears to have taken place in the spots that were not roughened. This behavior was observed in many other experiments on Au(111) in 0.1 M HClO_4_ (which has some small chloride contamination out of the bottle).^32^ Thus, we infer a correlation between the trace amount of chloride and the roughened areas on the sample. There is another somewhat puzzling anomaly in the behavior of the system with 1 μM chloride, which is the cycle dependence of the oxidation and reduction charge density. For 50 μM chloride, there is no cycle dependence (Figure 2c), in agreement with the lack of surface changes under those conditions. For 10 μM chloride, the oxidation/reduction charge density decreases with cycle number (Figure 4c). This is in qualitative agreement with our previous work (using 0 μM chloride) and was interpreted as the loss of (111) terraces during cycling (as Au(111) has the highest oxidation/reduction charge density). Remarkably, for 1 μM chloride, the oxidation/reduction charge density first decreases (signifying the loss of 111 terraces), then increases again (suggesting the formation of new 111 facets), and subsequently decreases again. We have no good explanation for this behavior at present, but it could be that we are seeing the superposition of two signals: one from the part of the surface that is roughening and another from the part that is not roughening. Future studies could examine whether trace amounts of oxygen in the electrolyte would influence the system in any way.

**Figure 8:**
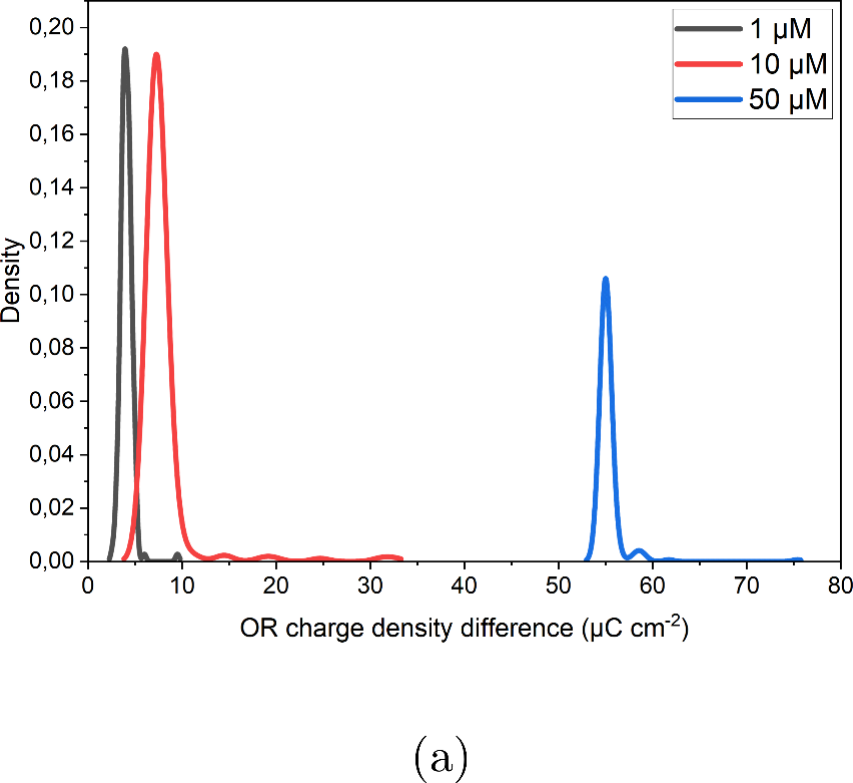
Histogram plot of the difference in the oxidation and reduction charge densities in 0.1 M H_2_SO_4_ and different concentrations of HCl.

## Conclusions

In this work, we performed an in situ EC-STM study of the evolution of a Au(111) electrode surface during 200 oxidation–reduction cycles (ORCs) in 0.1 M sulfuric acid with varying concentrations of HCl. The findings demonstrate how even a minor chloride concentration significantly alters the surface dynamics and surface roughening. Chloride ions rapidly dissolve Au atoms at their highest concentration (50 μM). Moreover, the high surface atom mobility under these conditions prevents the formation of detectable vacancy islands in the captured images during the cycles with only step line recession being observed. Consequently, only a few new step sites are generated, leading to minimal changes in the CVs and constant oxidation and reduction charge densities and the complete absence of surface roughening. The pronounced difference between the oxidation and reduction charges further proves the high dissolution rate of the Au atoms. At the lower chloride concentration (10 μM), the dissolution occurs at a significantly slower rate (given the comparison of the step line recession and disappearance of adatom islands), allowing the recognition of the initial step lines in the recorded image after multiple ORCs. The reduced atom mobility facilitates the imaging of vacancy islands formed by Au dissolution at terrace sites. These newly emerging step sites result in the appearance of two additional cathodic and anodic peaks in the recorded CVs. The oxidation and reduction charge densities exhibit a roughly logarithmic decay, resembling the behavior in pure sulfuric acid, indicating the inactivity of the new step sites in contributing to oxidation and reduction charges. Finally, at the lowest chloride concentration (1 μM), an apparent inhomogeneity in chloride adsorption was observed, leading to the formation of dark areas after the absorbed layer developed at positive potentials (0.9 V). These areas displayed distinct behavior: Au atom mobility was reduced, as indicated by the smaller island sizes, and the cycling caused no noticeable changes or roughening, even after 200 ORCs (in regions undisturbed by repeated scanning with the tip). The overall oxidation and reduction charge density exhibited multimodal behavior as a function of cycle number, the nature of which remains to be understood in detail.
